# The challenge of implant integration in partial meniscal replacement: an experimental study on a silk fibroin scaffold in sheep

**DOI:** 10.1007/s00167-018-5160-7

**Published:** 2018-09-27

**Authors:** Svenja Emmi Catherine Stein, Falk von Luebken, Daniela Warnecke, Cristina Gentilini, Nick Skaer, Robert Walker, Oliver Kessler, Anita Ignatius, Lutz Duerselen

**Affiliations:** 1 Institute of Orthopaedic Research and Biomechanics, Centre for Trauma Research Ulm, Ulm University Medical Centre, Helmholtzstraße 14, 89081 Ulm, Germany; 2 Department of Trauma and Orthopaedic Surgery, Hospital of the German Armed Forces Ulm, Oberer Eselsberg 40, 89081 Ulm, Germany; 3 Orthox Ltd., 66 Innovation Drive, Milton Park, Abingdon, Oxfordshire OX14 4RQ UK; 4 Centre of Orthopaedics and Sports, Albisriederstraße 243 A, 8047 Zurich, Switzerland

**Keywords:** Meniscus, Meniscectomy, Osteoarthritis, Meniscal replacement, Silk, Silk fibroin, Scaffold, Permanent replacement

## Abstract

**Purpose:**

To restore meniscal function after excessive tissue damage, a silk fibroin implant for partial meniscal replacement was developed and investigated in an earlier sheep model. After 6 months implantation, it showed promising results in terms of chondroprotection and biocompatibility. To improve surgical fixation, the material was subjected to optimisation and a fibre mesh was integrated into the porous matrix. The aim of the study was the evaluation of this second generation of silk fibroin implants in a sheep model.

**Methods:**

Nine adult merino sheep received subtotal meniscal replacement using the silk fibroin scaffold. In nine additional animals, the defect was left untreated. Sham surgery was performed in another group of nine animals. After 6 months of implantation macroscopic, biomechanical and histological evaluations of the scaffold, meniscus, and articular cartilage were conducted.

**Results:**

Macroscopic evaluation revealed no signs of inflammation of the operated knee joint and most implants were located in the defect. However, there was no solid connection to the remaining peripheral meniscal rim and three devices showed a radial rupture at the middle zone. The equilibrium modulus of the scaffold increased after 6 months implantation time as identified by biomechanical testing (before implantation 0.6 ± 0.3 MPa; after implantation: 0.8 ± 0.3 MPa). Macroscopically and histologically visible softening and fibrillation of the articular cartilage in the meniscectomy- and implant group were confirmed biomechanically by indentation testing of the tibial cartilage.

**Conclusions:**

In the current study, biocompatibility of the silk fibroin scaffold was reconfirmed. The initial mechanical properties of the silk fibroin implant resembled native meniscal tissue. However, stiffness of the scaffold increased considerably after implantation. This might have prevented integration of the device and chondroprotection of the underlying cartilage. Furthermore, the increased stiffness of the material is likely responsible for the partial destruction of some implants. Clinically, we learn that an inappropriate replacement device might lead to similar cartilage damage as seen after meniscectomy. Given the poor acceptance of the clinically available partial meniscal replacement devices, it can be speculated that development of a total meniscal replacement device might be the less challenging option.

## Introduction

Although it is well established that resection of meniscal tissue increases the risk for the development of osteoarthritis in the long term, more than 350,000 patients over the age of 35 years still receive partial meniscectomy in the US every year [18]. Damage or removal of meniscal tissue impairs load bearing inside the knee joint, since the menisci are responsible for the distribution of contact load over the articulating surfaces of femur and tibia. Consequently, there is a huge need for alternative treatment strategies, by which meniscal function can be restored, and thus, cartilage degeneration prevented. In recent years, the suitability of various artificial materials for meniscal replacement has already been investigated [1, 8, 17, 20, 25, 32]. Only two of these devices have been translated into clinical application (CMI^®^, Ivy Sports Medicine, Germany; Actifit^®^, Orteq Ltd., UK). However, their long-term chondroprotection has yet to be proven [17]. Moreover, there is no significant advantage of implanting these materials over meniscectomy, leading to a lack of acceptance among clinicians and limited use in clinical practice [17]. Due to intensive research in this field, there are further devices in preclinical development. Mimicking the collagen fibre orientation of the native meniscus might be a promising approach. NuSurface^®^ (Active Implants Israel Ltd., Israel) for total meniscal replacement has already proceeded to early clinical testing. The disc-shaped polycarbonate-urethane device is enforced by circumferentially running polyethylene fibres and is designed to be inserted into the knee joint without fixation to the tibia [4]. Balint et al. developed an absorbable collagen implant equipped with an anatomically placed network of degradable fibres (Meniscofix™, Novopedics Inc.) [1]. Special emphasis was put on the mechanical tensile behaviour of the total replacement device, as it should be able to convert axial loads into circumferential hoop stresses, thereby decreasing compressive loads on the tibial cartilage.

**Fig. 1:**
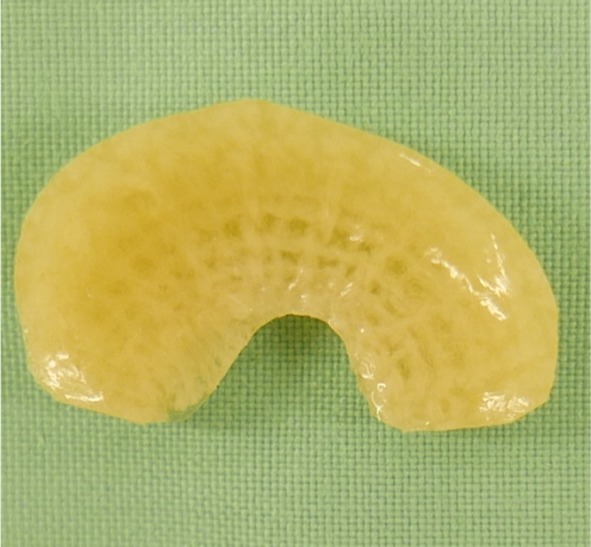
Top view of the anatomical (ovine) shaped silk fibroin scaffold for meniscal replacement. A horizontally running fibre mesh was integrated to improve surgical fixation

Recently, an alternative approach was implemented by the development of a permanent prosthesis for partial meniscal replacement. It is a silk fibroin scaffold made from *Bombyx mori* silkworm silk (FibroFix™, Orthox Ltd., Abingdon, UK). During the manufacturing process, fibroin is extracted and processed into a porous matrix. The first generation of implants was previously investigated in a sheep model [5, 6]. Thereby, partial meniscal replacement was carried out at the anterior horn of the medial meniscus, and after 6 months of implantation, it showed promising results in terms of biocompatibility and chondroprotection. However, fixation and integration into native meniscal tissue were insufficient, leading to dislocation of a minority of the implants. For a successful future application, improvements were necessary. Thus, the material was subjected to optimisation and a fibre mesh was integrated into the porous matrix, designed to improve secure anchoring in the remaining native meniscal tissue (Fig. 1). In the current study, fixation was further challenged by developing an ovine model which enabled a larger meniscal defect to be created than in the previous ovine meniscal replacement studies.

The aim of the current study was the in vivo evaluation of the second generation of silk fibroin implants in a sheep model. For this, a subtotal meniscal defect model was developed enabling replacement of a large proportion of the medial meniscus. After 6 months of implantation macroscopic, biomechanical as well as histological analyses were conducted. It was hypothesised that the integration of a fibre mesh into the replacement device leads to an improved surgical fixation to the remaining peripheral meniscal rim and consequently to enhanced integration into the host tissue and favourable chondroprotection.

## Materials and methods

Twenty-seven merino sheep (age: 2 ± 1 years, weight: 93 ± 8 kg) were randomly assigned to one of the three groups with nine animals each (Table 1). In groups 2 and 3, a large meniscal defect was created extending across the medial-posterior region representing a clinically highly relevant injury pattern [14]. Surgical access was developed to enable the implantation of a larger replacement device than in the previous partial meniscal replacement studies in the sheep. After 6 months of implantation, all animals were sacrificed and macroscopic, biomechanical as well as histologic evaluation of scaffolds and menisci as well as of the articular cartilage was conducted. In addition, India Ink staining of the articular surfaces was performed. The non-operated hind limbs always served as positive controls.

**Table 1:** Overview of the surgical groups and procedures

	Surgical procedure	Observation period (months)	*n*
Group 1	Sham surgery	6	9
Group 2	Subtotal meniscectomy	6	9
Group 3	Meniscus replacement by silk fibroin scaffold	6	9

### Silk fibroin scaffold

Natural silk fibres from the cocoon of the silkworm (*Bombyx mori*) were used as raw material for manufacturing of the silk fibroin scaffolds (FibroFix™, Orthox Ltd., Abingdon, UK). Due to the immunogenic potential of the sericin component of natural silk fibres [3, 30, 31], fibroin was extracted and processed into a porous matrix with the surface of the scaffolds being smooth [28]. During the optimisation process, direct light interference scanning of ovine menisci was performed and used to fabricate a prototype implant mould using three-dimensional print technology. This mould was subsequently used for creating the implants. As opposed to the first generation of silk fibroin scaffolds, the devices of the second generation are anatomically shaped, corresponding to the original shape of an ovine medial meniscus [5, 6]. Furthermore, a fibre mesh also fabricated from silk fibroin was integrated into the porous matrix to offer the potential for improved surgical fixation to the peripheral meniscal rim. The scaffolds were sterilised by gamma-irradiation and individually sealed in foil-laminate pouches containing sterile phosphate-buffered saline (PBS).

### Surgery

Surgery was performed under general anaesthesia and in supine position. A medial parapatellar arthrotomy including bony release of the femoral attachment of the medial collateral ligament was performed in all groups to gain access to the medial meniscus of the left stifle joint [5, 6]. Development of the surgical access allowed the complete meniscus to be exposed enabling creation of a large subtotal meniscal defect. In group 1 (*n* = 9), sham surgery was carried out without procedure on the medial meniscus (sham group). In groups 2 (meniscectomy; *n* = 9) and 3 (meniscal replacement; *n* = 9), subtotal meniscectomy of the medial meniscus was conducted leaving only a 2 mm-meniscal rim as well as the anterior and posterior horns intact (Table 1). This large defect was left empty in group 2. In group 3, subtotal meniscal replacement using the silk fibroin scaffold was performed. Thereby, the resected part of the medial meniscus served as a template to cut the device to the correct size and shape. For fixation of the silk fibroin implant, three horizontal mattress sutures, which did not penetrate the surface of the device, were tied at the periphery of the meniscus. The medial collateral ligament was re-attached to the femur using a claw plate and bicortical screw fixation. Afterwards, the joint capsule was sutured and the surgical incision was closed in layers. The sheep were allowed unrestricted movement and full weight bearing immediately after surgery. Antibiotic and anti-inflammatory medication was administered over three consecutive days. After 6 months, all animals were sacrificed.

### Macroscopic evaluation

Both hind limbs of all sheep underwent macroscopic evaluation. The knee joint region was examined for any signs of inflammation or impairment of wound healing. The integrity and shape of the scaffold as well as the extent of integration into the native meniscal rim was examined. Finally, a semi-quantitative evaluation of the articular surfaces of femur and tibia was conducted using a modified grading score according to Outerbridge [15].

### Biomechanical evaluation

To determine the biomechanical properties of the silk fibroin scaffold in comparison with native meniscal tissue, a compression stress-relaxation test was performed as previously described [28]. Therefore, two cylindrical samples (*∅* = 4.6 mm) were cut out of each scaffold (*n* = 10) using a biopsy punch. In the sham group, one sample of the anterior (*n* = 9) as well as of the posterior (*n* = 8) part of the medial meniscus of the operated knee joint were punched out, respectively. Medial meniscus samples of the non-operated hind limbs of both groups served as control (*n* = 34). Testing was conducted according to a modified testing protocol published earlier [28]. The equilibrium modulus *E*_eq_ (MPa) was calculated, which was defined by the stress at equilibrium ${\sigma _{t \to \infty }}$ (averaged over the last 10 min of testing) divided by the applied constant strain *ε*:

1 $${E_{{\text{eq}}}}=~\frac{{{\sigma _{t \to \infty }}}}{\varepsilon }~;~\;\varepsilon =0.12.$$

To additionally assess potential material changes of the silk fibroin scaffolds over the implantation period, the obtained data were compared to the mechanical properties of the scaffold before implantation, which were determined by Warnecke et al. using the same testing protocol [28].

For the biomechanical evaluation of articular cartilage, an indentation test on the surface of the medial tibial condyle of the operated as well as of the non-operated hind limbs (each *n* = 7, group 3: *n* = 6) was performed as previously reported [5, 6]. Indentation testing was conducted at three different measuring points (MP) for every medial tibial condyle (Fig. 5). Strain (*ε*) at maximum force was held constant for 20 min. The equilibrium modulus *E*_eq_ was calculated by the stress at equilibrium $\left( {\frac{{{F_{{\text{eq}}}}}}{A}} \right)$ divided by the strain at equilibrium $\left( {\frac{{\Delta {l_{{\text{eq}}}}}}{{{h_0}}}} \right)$:

2 $${E_{{\text{eq}}}}=\frac{{{F_{{\text{eq}}}}}}{A}~ \times \frac{{{h_0}}}{{\Delta {l_{{\text{eq}}}}}}.$$

Measurements and data acquisition during biomechanical testing were carried out using the standard software by Zwick/Roell (TestXpert II, Zwick GmbH, Germany). Afterwards, the data were analysed using MATLAB^®^ R2016a (MathWorks, Natick MA, USA).

After biomechanical testing, samples were fixed in formaldehyde (4%) for histologic sample preparation.

### India Ink staining

Femoral and tibial condyles of the operated as well as of the non-operated control knees (each *n* = 54) were subjected to India Ink staining, binding ink particles only to softened and fibrillated articular cartilage [5, 6]. By image analysis (Photoshop CS 4, Adobe Systems, San José, USA) and a custom-made software routines (MATLAB^®^ R2016a, MathWorks, Natick MA, USA), the percentage of black stained area was determined.

### Histological evaluation

For histological evaluation, samples of synovial membrane, scaffold, and native meniscal tissue of the operated as well as of the non-operated hind limb were stained with Haematoxylin and Eosin (HE) and analysed qualitatively using an optical microscope (DMI6000B, Leica Mikrosysteme Vertrieb GmbH, Wetzlar, Germany). Thereby, synovial membrane of all animals was examined with regard to structural changes, i.e., hyperplasia and hypertrophy of synovial cells as well as villous formation. In addition, inflammatory cells in terms of synovial giant cells were assessed. The scaffold was examined with respect to integrity, cell infiltration, and subsequent tissue deposition and overall integration into native tissue.

Articular cartilage samples of the medial tibial plateau were stained with Safranin-O/Fast Green and examined using a photo microscope (Axiophot 451887, Zeiss, Germany). For semi-quantitative assessment of articular cartilage condition, a grading scheme according to Mankin et al. was applied [12].

The study was approved by the Regulatory Authority in Tübingen, Germany (registration number 1229). All animal procedures were carried out according to the national and international regulations for the care and use of laboratory animals.

### Statistical analysis

To calculate sample size, a power analysis based on the results of the previous study by Gruchenberg et al. was performed [5, 6]. The required group size of eight animals was increased to account for a possible loss of animals during surgery and in the course of the study. Consequently, nine animals were used in each surgical group. Statistical analysis of the data was carried out using Graph Pad^®^ Prism (Graph Pad Software Inc., La Jolla, USA). After normal distribution was checked (D`Agostino and Pearson Omnibus test, Shapiro–Wilk test, and Kolmogorov–Smirnoff test), mean ± standard deviation was determined. Data with small sample size (*n* < 8) were expressed as single values with median, minimum, and maximum. Differences within groups (operated limb vs. non-operated control) as well as between groups regarding the macroscopic evaluation (outerbridge grading), India ink staining and biomechanical properties of articular cartilage were evaluated using a two-way analysis of variances (ANOVA) for repeated measures with post hoc test (Šidák-/Tukey test).

A one-way ANOVA with post hoc Fisher’s LSD test was conducted, to compare the data of the biomechanical analysis of scaffold and meniscus. Significance was always set at *p* ≤ 0.05. Data from the histological analysis were assessed descriptively.

## Results

### Clinical observations

All sheep recovered well after surgery and there were no wound healing disorders. By 3 day post-surgery, all animals showed a normal gait pattern which was maintained throughout the in-life phase.

### Macroscopic evaluation

**Fig. 2:**
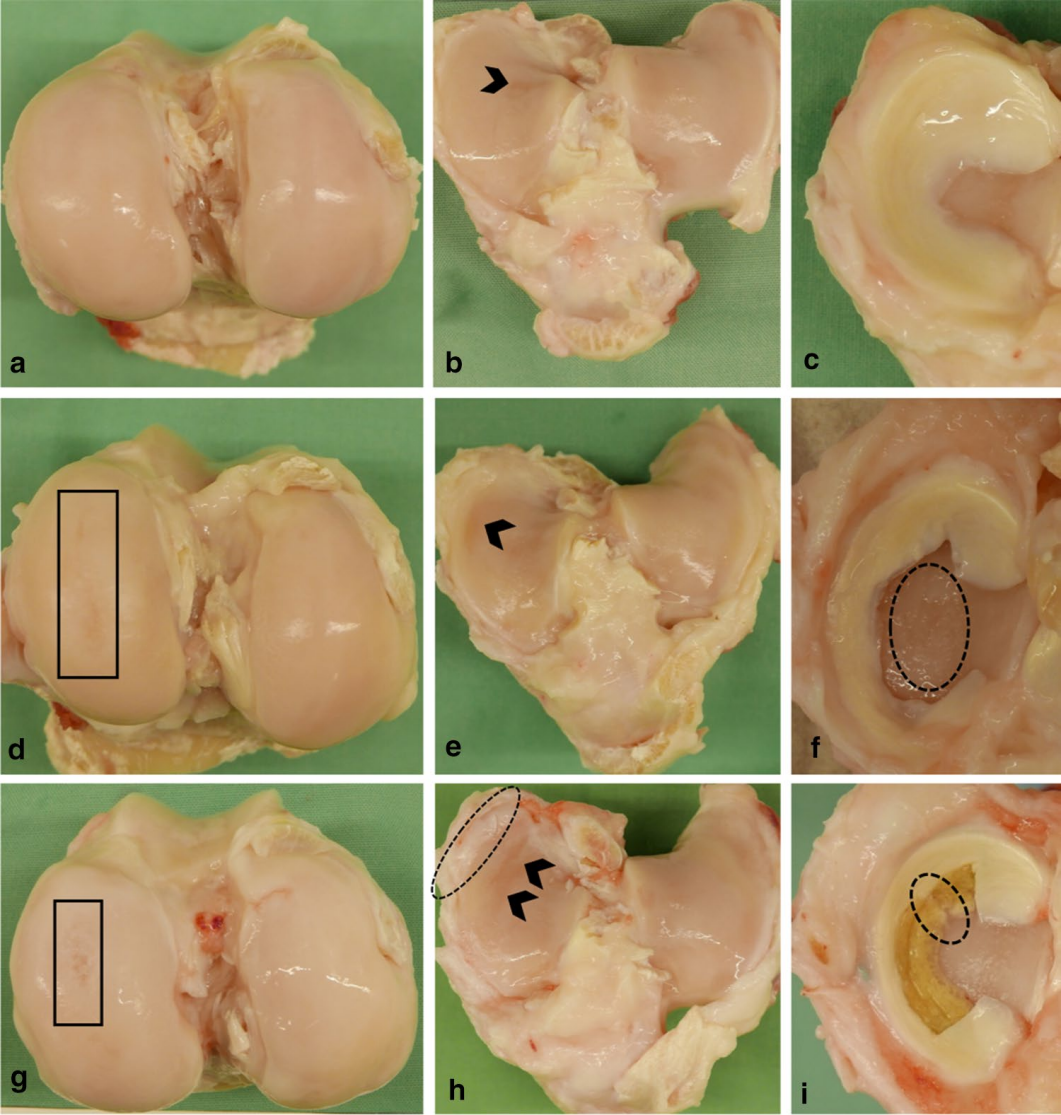
Representative photographs of operated joints of the sham- (**a**–**c**), meniscectomy- (**d**–**f**), and implant group (**g**–**i**). **a** Femoral cartilage without surface roughening after sham surgery. **d, g** Femoral cartilage displaying elongated surface roughening over the articulating area (black boxes) after meniscectomy and meniscal replacement. **b** Softening of the tibial cartilage, limited to the *Eminentia intercondylaris* (black arrowhead). **e, h** Extended softening of medial tibial cartilage (black arrowheads) after meniscectomy and meniscal replacement, including broadening of the joint surface in the implant group (dotted line). **c** Physiological medial meniscus of a sham animal. **f** Large subtotal meniscal defect and considerable cartilage erosion (dotted line). **i** Silk fibroin implant for subtotal medial meniscal replacement, displaying a radial rupture at the transition between the middle and posterior zone (dotted line)

In the area of surgical access, there was a moderate fibrosis of the subcutaneous tissue as well as a fibrotic thickening of the joint capsule. There were no signs of inflammation of the operated knee joints. No tissue regeneration was visible inside the meniscal defects in the meniscectomy group (Fig. 2f).

**Fig. 3:**
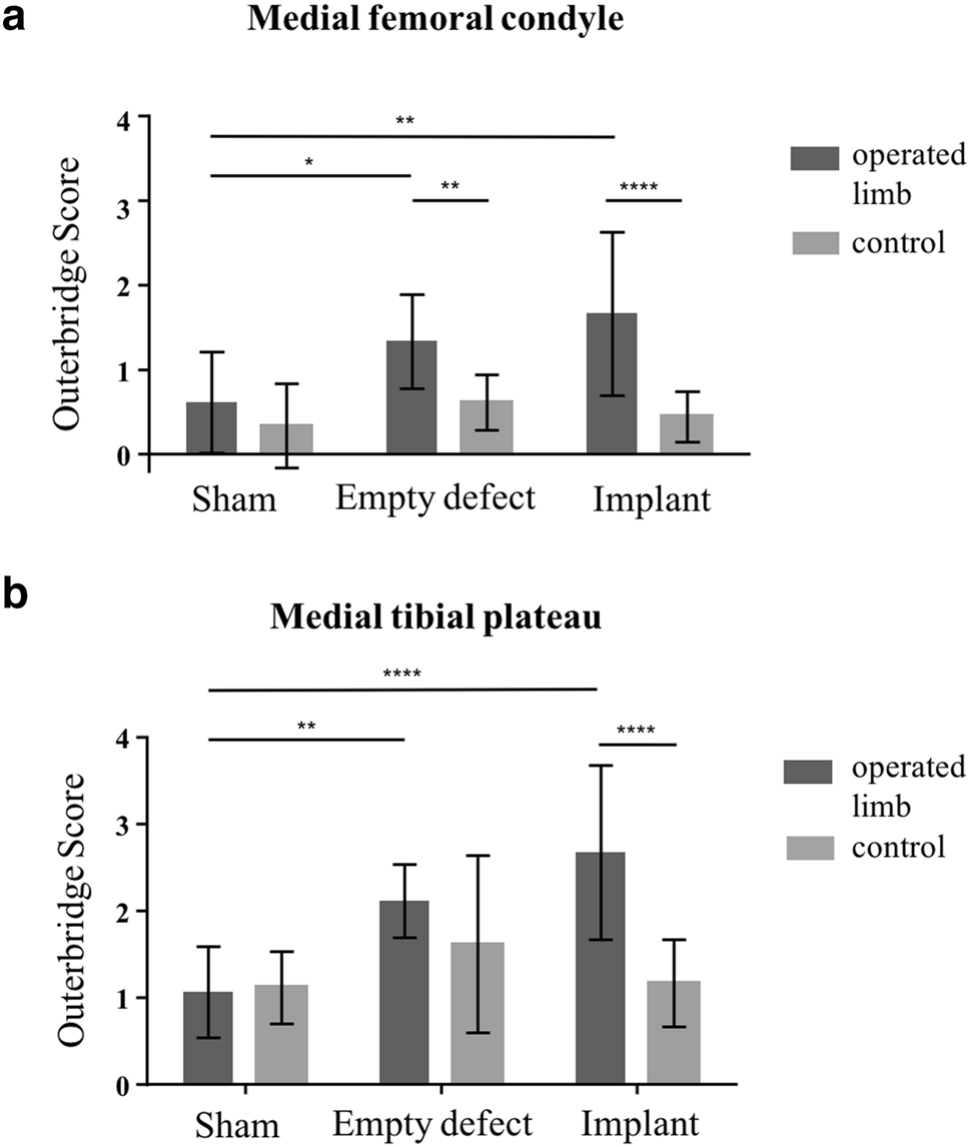
Classification of articular cartilage damage according to Outerbridge [15]. **a** Outerbridge scores of medial femoral condyles and **b** of medial tibial condyles of operated joints and non-operated controls (0 = no visible alterations). Mean ± standard deviation; *p < 0.05; **p < 0.01; ****p < 0.0001

In the implant group, the scaffold material was deteriorated and only partially located in the defect in three of nine animals, whereas in six sheep, the scaffold was located in the defect post mortem. However, three of these devices displayed a radial tear at the transition between the middle and posterior zone (Fig. 2i). In general, there was no solid tissue connection between the implant material and the remaining meniscal rim. Regarding articular cartilage condition, semi-quantitative grading according to Outerbridge revealed that there was no difference in cartilage condition between the meniscectomy- and implant group, both displaying significantly more cartilage degeneration compared to the sham group (femur: *p* < 0.05, respectively; tibia: *p* < 0.01, respectively) (Figs. 2, 3). However, slight degeneration of the articular surface at the area of the *eminentia intercondylaris* could also be detected in the sham group. In the meniscectomy- and in the implant group, there was also a significant difference between the operated and non-operated hind limbs regarding femoral articular cartilage condition (*p* < 0.01, respectively). The tibial plateau of the non-operated hind limbs showed considerable cartilage degeneration in all groups. However, there was significantly more cartilage degeneration after meniscal replacement compared to the non-operated limb of these animals (*p* < 0.001). Furthermore, there was a slight osteophytic growth at the medial femoral condyle in one sheep of the meniscectomy group as well as at the medial tibial plateau in two and six sheep of the meniscectomy- and implant group, respectively (Fig. 2h).

### Biomechanical evaluation

**Fig. 4:**
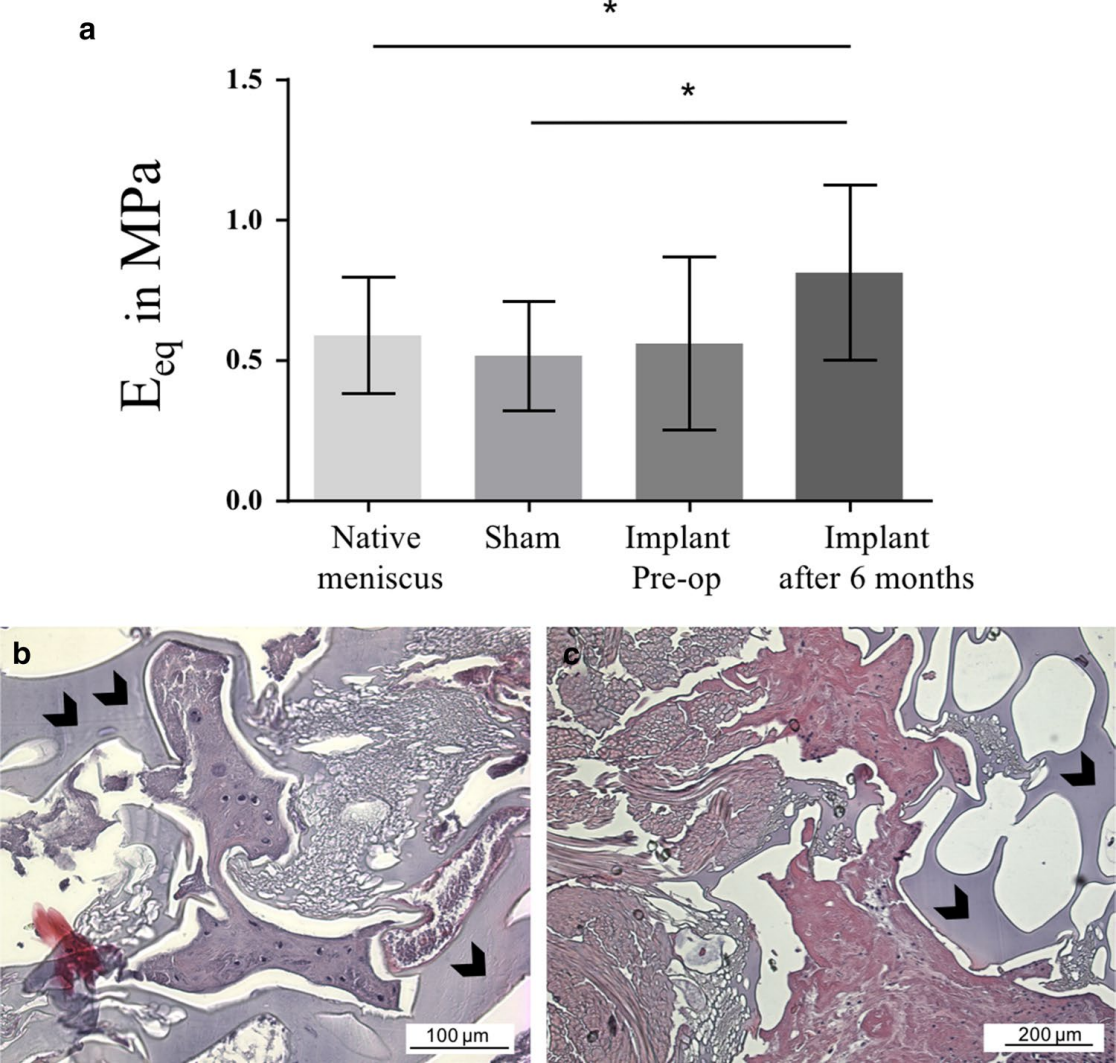
Results of the biomechanical and histological evaluation of the silk fibroin implant. **a** Equilibrium modulus *E*_eq_ in MPa (mean ± standard deviation) of the silk fibroin implant pre- and post 6 months of implantation in comparison with native meniscal tissue of the non-operated limb as well as of the sham group. *p < 0.01. **b** Histological section of the silk fibroin implant. Peripheral tissue infiltration with few chondrocyte-like cells; 200 ×-magnification. **c** Disorganised tissue infiltration; 10 ×-magnification. Black arrowheads: silk fibroin device

Compared to the mechanical properties of the silk fibroin material before implantation determined by Warnecke et al. [28], compression stress-relaxation test revealed that the equilibrium modulus *E*_eq_ of the implant increased during implantation (Fig. 4a). After 6 months, the implant was significantly stiffer than the native medial meniscus in the sham- (0.5 ± 0.2 MPa; *p* < 0.01) and control group (0.6 ± 0.2 MPa, *p* < 0.01). However, there was no significant difference between the equilibrium modulus *E*_eq_ of the implant before implantation and the native medial meniscus of the sham and control groups.

**Fig. 5:**
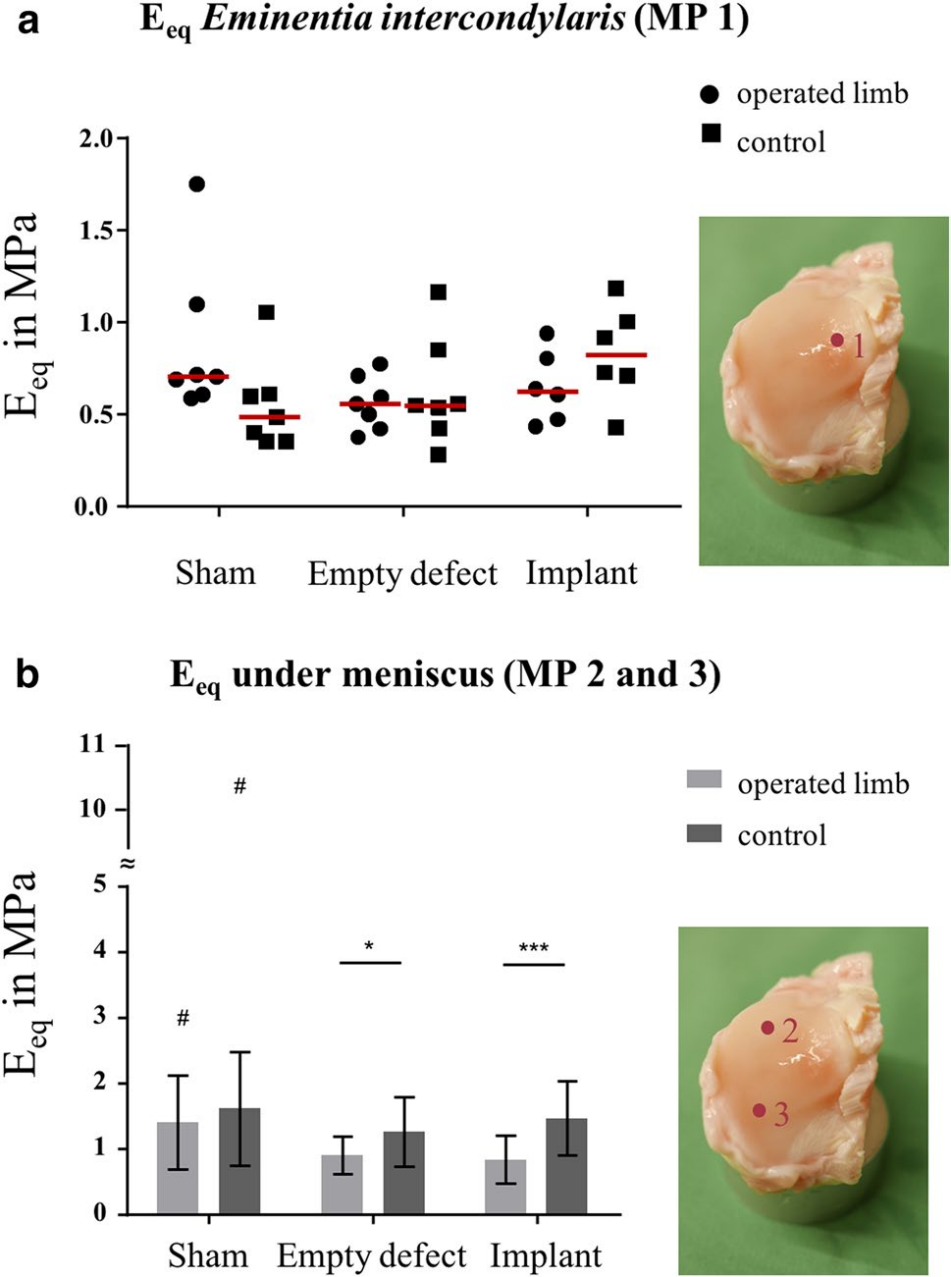
Biomechanical evaluation of tibial cartilage. **a** Equilibrium modulus *E*_eq_ in MPa (median and single values) of tibial cartilage at the *Eminentia intercondylaris* (MP 1) and **b** under the meniscus (MP 2, 3). *p < 0.05; ***p < 0.0001; ^#^statistical outlier

Regarding the biomechanical evaluation of the tibial cartilage at three different measuring points, softening of articular cartilage was more pronounced at MP 1 located at the *Eminentia intercondylaris* compared to MP 2 and 3, located under the meniscus/defect/implant (Fig. 5). Regarding the equilibrium modulus (*E*_eq_) at MP 1, there were no differences between the groups (Fig. 5a). This was true for the operated hind limbs as well as for the non-operated controls. At MP 2 and 3, there was a significant softening of tibial cartilage after 6 months in the meniscectomy- and implant group (*p* < 0.05) (Fig. 5b).

### India Ink staining

**Fig. 6:**
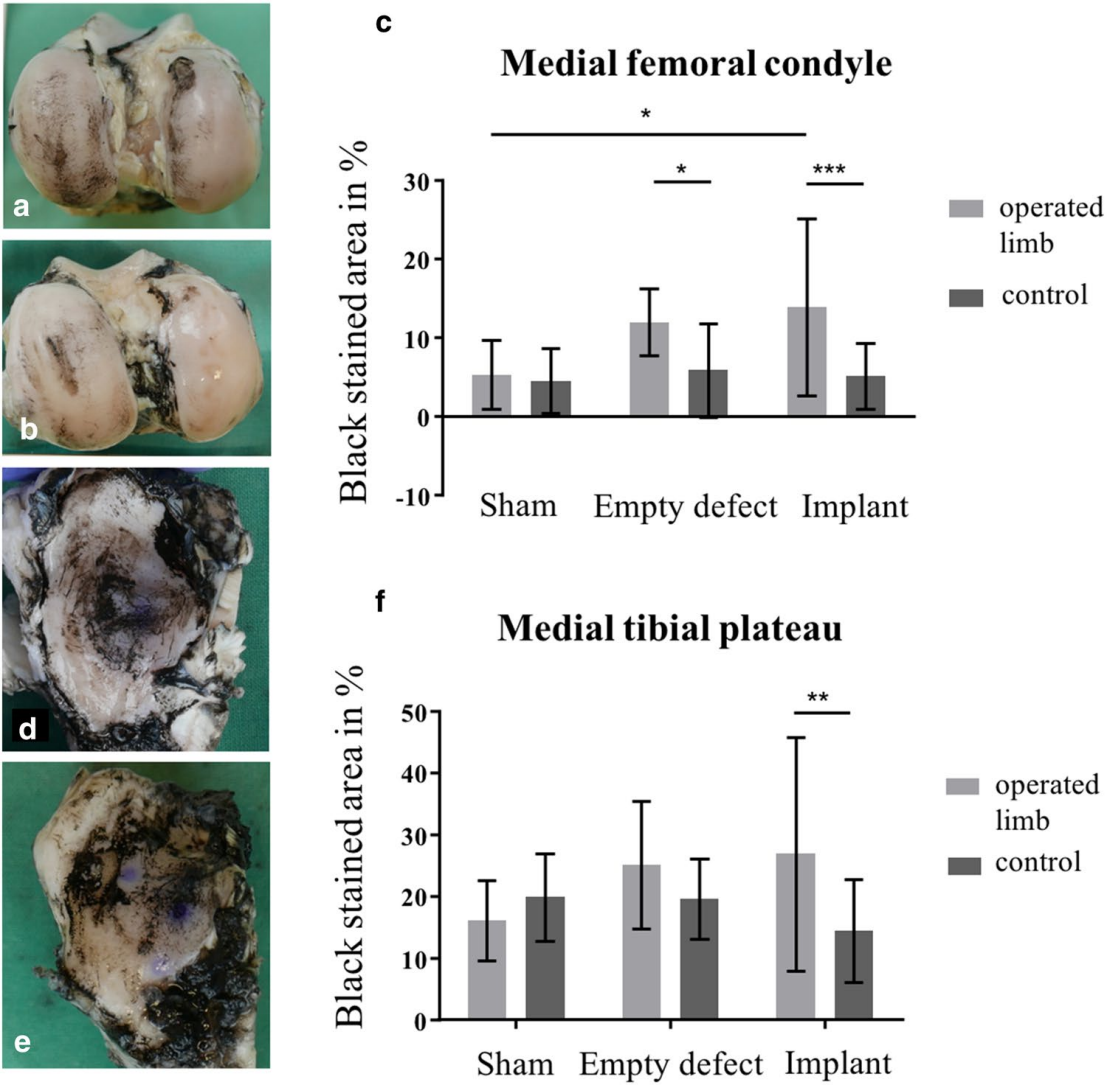
India ink staining of the articular surfaces of femur and tibia. **a** Femoral condyles of an animal of the meniscectomy group, displaying considerable staining at the medial femoral condyle. **b** Femoral condyles of an animal of the implant group. **c** Percentage (%) of black stained area at the medial femoral condyle. Mean ± standard deviation; *p < 0.05; ***p < 0.001. **d** Medial tibial plateau of an animal of the meniscectomy group, displaying extended fibrillation of the cartilage surface. **e** Medial tibial plateau of an animal of the implant group. **f** Percentage (%) of black stained area at the medial tibial plateau. Mean ± standard deviation; **p < 0.01

By India Ink staining of the medial articular surfaces of the femur and tibia, macroscopic Outerbridge grading could be confirmed. Compared to the sham group, cartilage degeneration of femur and tibia was more pronounced in the meniscectomy- and implant groups (Fig. 6a, b, d, e). Thereby, meniscectomy and meniscal replacement led to a significant deterioration of the femoral cartilage (*p* < 0.05).

### Histological evaluation

Hypertrophy as well as hyperplasia of synovial cells was the most abundant change of the synovial membrane, occurring in operated as well as non-operated joints of individual animals in all groups. More pronounced structural changes of the synovial membrane by villous proliferation occurred in the operated joint of three sheep of the meniscectomy group. This was by trend more than in the other groups. However, single inflammatory cells in the form of synovial polynuclear giant cells were detected in four animals after meniscal replacement, which was slightly more than in other groups. In the sham and meniscectomy groups, giant cells were identified in only one animal.

Regarding meniscal tissue integrity in the sham group, there were no differences in staining as well as structural characteristics between the operated and non-operated joints. Whereas after meniscectomy, the edges of the untreated meniscal defects exhibited considerable loss of structure in most animals. Furthermore, cluster of chondrocytic cells was detected around the artificial defect in five sheep and three of them additionally displayed basophilic regeneration tissue. Due to a lack of sufficient tissue integration, implants were evaluated independently from the remaining native meniscal tissue, as they were detached during sample preparation. Two sheep were excluded from evaluation, because the implant was completely dislocated in vivo. In all histological samples of the replacement device, the macroporous structure of the implant was clearly visible. The peripheral pores of the matrix were filled with fibrin-like tissue and few chondrocytic cells in five sheep (Fig. 4b, c). In two of those implants, the infiltrating tissue was more organised with the beginnings of fibrous organisation. However, in one device there was only amorphous tissue, whereas another device was completely empty.

**Fig. 7:**
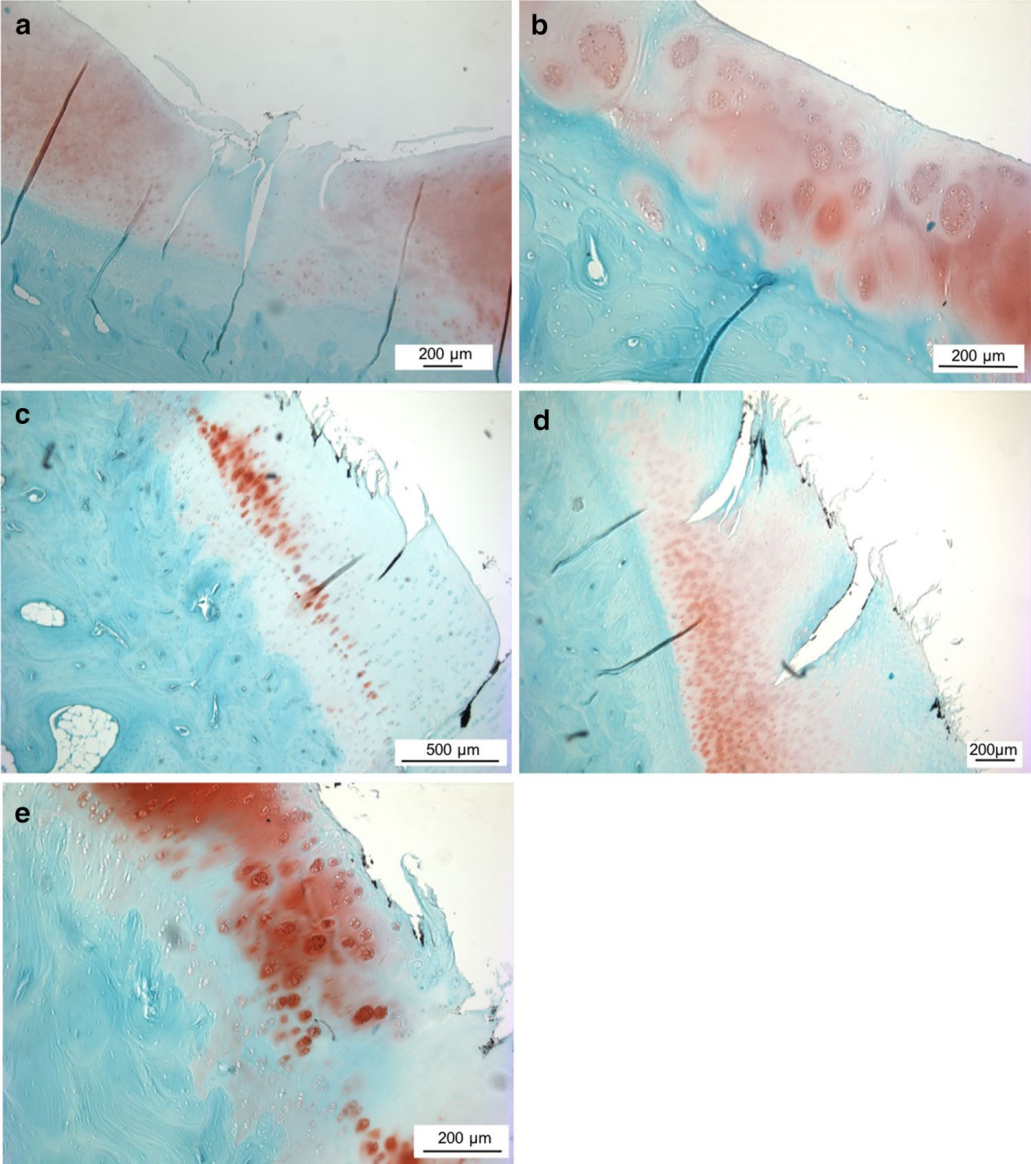
Histological images of the articular cartilage of the medial tibial plateau. **a** Histological section of the medial tibial plateau of an animal of the meniscectomy group, displaying a considerable reduction in safranin-o staining intensity, fissures down to the calcified cartilage as well as diffuse hypocellularity. 50 ×-magnification. **b** Magnification of **a**. Clusters of chondrocytes. 100 ×-magnification. **c** Tibial cartilage after meniscal replacement, showing considerable loss of staining intensity as well as reduction in cellularity. 50 ×-magnification. **d** Fissuring of cartilage surface and cracks into the radial zone of articular cartilage after meniscal replacement. 50 ×-magnification. **e** Magnification of **c**. Chondrocytes arranged in clusters. 100 ×-magnification

**Fig. 8:**
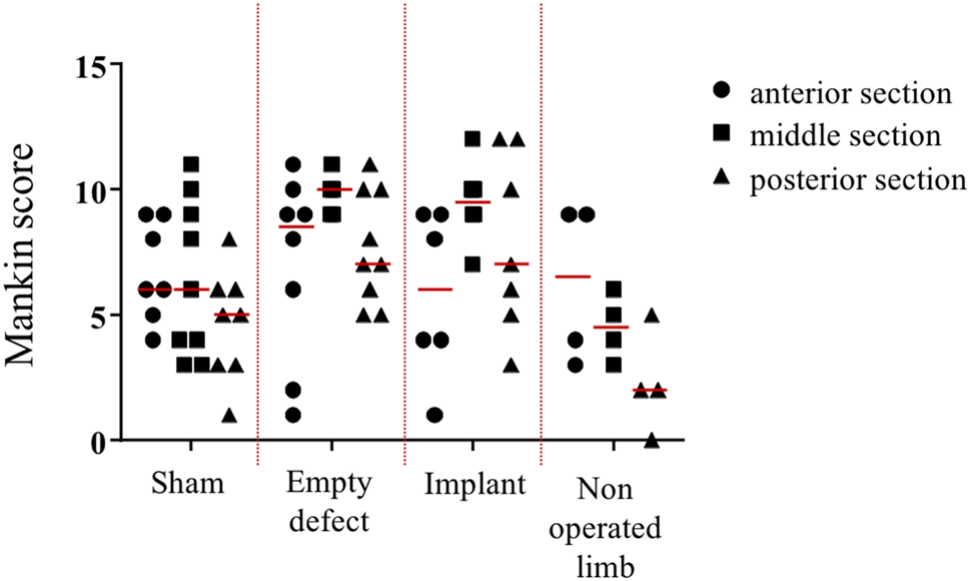
Overview of the degeneration grade according to Mankin at different locations of the tibial plateau

Histological sections of the anterior, middle, and posterior aspect of each medial tibial plateau were subjected to semi-quantitative histological classification using a grading scheme according to Mankin [12]. In the sham group, there were no differences between the individual aspects regarding the total degree of degeneration. Structural changes of articular cartilage remained limited to the area of the *Eminentia intercondylaris*. Deterioration of tibial cartilage structure was more pronounced after meniscectomy, extending over the whole articular surface (Figs. 7a, b, 8). Thereby, especially the middle area of the tibial plateau displayed excessive degenerative changes. The tidemark was disrupted in the middle and posterior sections of most animals. In the implant group, cartilage sections displayed a high variability in the total degree of degeneration (Figs. 7c–e, 8). As in the meniscectomy group, particularly the middle section of the tibial plateau was affected. Cartilage damage was not restricted to central areas close to the *Eminentia intercondylaris*, but involved peripheral cartilage areas as well. The cartilage tidemark was disrupted in middle and posterior sections in nearly all animals. Tibial cartilage sections of contralateral non-operated control joints showed similar results compared to the sham group, thereby structural changes of cartilage were largely restricted to superficial surface irregularities.

## Discussion

The most important finding of the present study was that the hypothesised enhanced integration into the host tissue and favourable chondroprotection by an improved surgical fixation of a partial meniscal replacement device to the remaining peripheral meniscal rim was only partially confirmed. Although surgical fixation was improved, after 6 months of implantation, softening of femoral and tibial cartilage of the medial joint compartment was observed macroscopically, mechanically as well as histologically. Thereby, no differences to untreated meniscal defects were detected. Furthermore, some of the implants were partially destroyed. Similarly, two other studies reported pronounced destruction of implants in the posterior aspect of the medial compartment [9, 26, 27]. Although one of these studies was carried out in goats, a similar mechanism can be anticipated due to the same anatomy. In the medial compartment of the ovine knee joint, there is a significantly higher contact area compared to the lateral compartment [10]. Accordingly, 60–80% of the total joint force, corresponding to about 1.7 times BW, are transmitted solely through the medial joint [22]. Thereby, peak loads occur in the posteromedial aspect of the joint, probably contributing to the observed scaffold tearing. The development of functional meniscal replacement withstanding these high joint forces is very challenging particularly in our paradigm in which a larger meniscal replacement was used requiring the implant to resist a proportionally greater amount of force.

The equilibrium modulus *E*_eq_ of the silk fibroin implant before implantation, determined by Warnecke et al. in an unconfined compression relaxation test according to Chia et al., was not significantly different from native ovine meniscal tissue [2, 28]. Nevertheless, during implantation, the stiffness of the implant material increased, leading to a significant difference compared to native ovine meniscal tissue after 6 months. The increased stiffness might have prevented successful integration into native tissue as discontinuity of stiffness across the meniscus–scaffold interface is likely to cause relative movements too high for the initiation of a solid tissue connection. This effect may have been magnified by the larger size of the implant in comparison with the previous studies and the unrestricted movement of the sheep immediately post-implantation. As stated in the basic requirements for meniscal replacement, the mechanical properties of a replacement device should resemble native meniscal tissue as close as possible to allow for successful integration and protection of the articular surfaces [17]. Sandmann et al. showed that the biomechanical properties of two clinically available artificial devices for meniscal replacement (CMI^®^ and Actifit^®^) were significantly inferior compared to human meniscal tissue [19]. However, material properties of the constructs may change with proceeding tissue infiltration and scaffold matrix degradation in vivo. Indeed, investigations of early material variants of the Actifit^®^ scaffold in a dog model revealed that the initial material stiffness increased with progressing implantation time, but even after 6 months still considerably lower compared to native meniscal tissue [23, 24]. Thus, cartilage degeneration could not be prevented after total meniscal replacement. In addition, a longer follow-up study over 24 months of implantation again revealed comparable cartilage damage in the meniscectomy- and implant group [29]. Hence, the mechanical properties directly after implantation might have been too weak to effectively participate in load transmission, contributing to mechanical overloading and early cartilage degeneration. To check the mechanical properties of the silk fibroin implant against the commercially available scaffolds, an indentation test was performed, using the testing protocol according to Sandmann et al. [28]. Notably, the silk fibroin implant displayed superior initial mechanical properties compared to CMI^®^ and Actifit^®^, exhibiting high mechanical competence prior to implantation.

In the previous studies by our own research group as well as by another group, the mechanical properties of meniscal replacement devices before and after implantation were investigated [5, 6, 13, 16]. Gruchenberg and colleagues evaluated the performance of the first generation of silk fibroin implants for partial meniscal replacement in the sheep model [5, 6]. Similar to the current study, however, there was only limited tissue infiltration of the porous matrix. Thus, our hypothesis that the integration of the fibre mesh would lead to an improved integration into native tissue could not be confirmed in the current study. Two other studies investigated the mechanical properties of a device for total meniscal replacement at different time points [13, 16]. The implant consisted of collagen and hyaluronic acid and was reinforced by circumferential fibres, thereby mimicking native meniscus structure (Meniscofix™, Novopedics Inc.). Over 16 and 32 weeks of implantation, mechanical properties increased considerably compared to the baseline condition of the implant. However, only 25–40% of the stiffness of native meniscal tissue were attained [13]. Nevertheless, articular cartilage integrity was superior compared to the meniscectomised control joints, indicated by a decreased Mankin score as well as percentage of damaged area. After 52 weeks of implantation, further increase of the material properties was detected and chondroprotection was maintained. The increased mean equilibrium modulus represents about 51% of the stiffness of native meniscal tissue [16]. Main reasons for the increased mechanical performance could be the confirmed cell infiltration as well as a hypothesised remodelling process of the tissue due to mechanical stimulation.

Due to different mechanical test setups among the studies, direct comparison of the mechanical properties of different materials is not possible. To the best of our knowledge, the silk fibroin implant is the only scaffold with initial mechanical properties that closely resemble native meniscus tissue, thereby most likely meeting the requirements for meniscus replacement. It should be noted that replacement devices, displaying an inadequate mechanical competence might not be able to prevent early cartilage degeneration in vivo. Conversely, a material that is too stiff may lead to the direct deterioration of the cartilage surface [17]. For the silk fibroin scaffold evaluated in the current study, the increase in stiffness during implantation cannot be attributed to cell infiltration with subsequent matrix deposition, as there was only limited cell and tissue infiltration detected in peripheral pores of the implants. The increase in stiffness may rather be due to a closer alignment of fibroin molecules, exclusion of water from the pore walls, increased hydrogen bond formation as well as beta-formation in the protein nanofibrillar structure as a possible consequence of cyclic compression inside the joint. Comparable increases in stiffness were observed over the first million load cycles when scaffolds have been fatigue tested through 5 million load cycles in vitro (personal communication). Improvements regarding preservation of the promising initial mechanical properties of the silk fibroin scaffold over the whole implantation period are necessary.

In general, meniscal replacement is further complicated in case of partial defects, where an alloplastic material has to integrate into native tissue. As already mentioned, to allow for a solid connection between a biomaterial and the tissue to be replaced, comparable mechanical features are mandatory. However, the specific properties of the meniscus, i.e., anisotropy and inhomogeneity, are difficult to imitate with an alloplastic material. Distinct relative movements at the tissue interface and high mechanical loads within the posterior aspect might have hampered integration of the silk fibroin scaffold in the current study. One could speculate that the requirements of high mechanical competence and sufficient flexibility to adapt to the host tissue under varying motion and loading conditions contradict each other. This makes permanent partial meniscus replacement an exceptionally hard challenge to achieve. Referring to the study by Patel et al., it could be speculated that a successful integration might be more important than to fully restore the mechanical properties of the native meniscus. However, the minimum level of mechanical properties, necessary for a satisfactory meniscal function remains unknown. To allow for tissue infiltration, mechanical properties should resemble native tissue as closely as possible. This is especially true for large meniscal defects such as were created in this study. As it is questionable if the specific properties of meniscal tissue, i.e., inhomogeneity and anisotropy, can be imitated by an alloplastic material, development of a total meniscal replacement device might be the less challenging option.

The fact that all sheep returned to full weight bearing immediately after surgery is a major limitation of the current study, as significant movement of the meniscus during flexion and extension might have produced excessive stress on the interface between implant and host tissue and consequently impaired tissue regeneration and subsequent integration of the devices. However, to account for this, the sheep were housed in small pens for the first weeks after surgery to limit post-operative movement. Furthermore, there is literature questioning a positive influence of post-operative immobilisation on meniscal healing [7]. In this context, it should also be mentioned that resection of meniscal tissue was probably not executed into the vascularised zone of the meniscus as indicated by missing tissue regeneration in the meniscectomy group, usually reported in sheep [11]. Insufficient connection to the vascular supply of the meniscus will impede tissue ingrowth into the implant. It can be assumed, that in sheep the vascularized area includes only 1 mm of the meniscal rim [21]. However, leaving a 1 mm peripheral meniscal rim might be insufficient for adequate fixation of the implants and the prevention of extrusion.

Partial meniscal replacement remains extremely challenging as adequate mechanical strength together with high flexibility are requirements not accomplishable yet. Given the poor acceptance of the clinically available partial meniscal replacement devices, it can be speculated that development of a total meniscal replacement device might be the less challenging option.

## Conclusions

Although surgical fixation of the silk fibroin implants was improved, there was neither evidence of significant integration with meniscal host tissue nor satisfactory chondroprotection in a sheep model over 6 months of implantation. Biocompatibility of the silk fibroin scaffold could be reconfirmed in the current study; however, the silk fibroin is not suitable for use at the current stage.

## Abbreviations

PBS: Phosphate-buffered saline
MP: Measuring points
HE: Haematoxylin and eosin
ANOVA: Analysis of variances
BW: Body weight
